# Fusion or Gemination? Diagnosis and Management in Primary Teeth: A Report of Two Cases

**DOI:** 10.1155/2021/6661776

**Published:** 2021-06-01

**Authors:** Mouna Ben Salem, Farah Chouchene, Fatma Masmoudi, Ahlem Baaziz, Fethi Maatouk, Hichem Ghedira

**Affiliations:** ^1^Pediatric and Preventive Dentistry Department, Faculty of Dental Medicine of Monastir, Monastir, Tunisia; ^2^ABCDF Laboratory of Biological, Clinical and Dento-Facial Approach, University of Monastir, Monastir, Tunisia

## Abstract

Primary double teeth (PDT), referring to either gemination or fusion, are one of the most important and frequent developmental dental anomalies that can affect children's oral health. Many clinical complications are correlated with these anomalies, such as dental caries, malocclusions, periodontal problems, and dental anomalies in permanent dentition. The aim of this report was to describe, through two cases, a case of gemination and another of fusion, the clinical management, the consequent effects, and the possible repercussions of these two developmental abnormalities on permanent successors. The first case involved gemination of the primary maxillary left central incisor (#61) in a 6-year-old boy. The patient presented with dental caries in the geminated tooth and its contiguous primary left lateral incisor (#62). The radiological examination revealed a slight developmental delay in the permanent left lateral incisor (#22). The treatment plan involved performing a pulpotomy and restoring the dental crowns of the affected teeth. The second clinical case describes a 6-year-old girl with unilateral fusion between a primary mandibular lateral incisor (#72) and a supernumerary tooth (#72'). The treatment included restoration of the deep grooves of the fused teeth using flowable composite and coronal restoration of the contiguous left primary canine (#73). Clinical and radiological follow-ups were performed every 3 months to monitor the development of teeth. No clinical and radiological symptoms were noted during the follow-up visits. PDT may require a multidisciplinary treatment. They should be diagnosed early to avoid and treat any possible complications in both the primary teeth and their permanent successors.

## 1. Introduction

Developmental dental anomalies may include abnormalities in teeth number, size, shape, structure, and position [1]. Only few studies have reported the frequency of these developmental dental hard tissue anomalies in primary dentition. As reported in the literature, their prevalence ranges from 0.5% to 7% [2]. However, Folayan et al. [1] found a prevalence of 8.2% in 3 to 5-year-old Nigerian children in 2019.

Primary double teeth (PDT), often referring to both fusion and gemination, are described as one of the most frequent developmental dental anomalies in primary dentition [2–4].

Unlike tooth fusion which is defined as the union between the enamel or both dentin and enamel of two or more separate developing tooth buds [5], gemination is known as an attempt of a single tooth bud to divide. Fusion has usually been confused with gemination, especially if it includes a supernumerary tooth [6]. Primary fused teeth (PFT) are more frequently observed in primary than in permanent dentition [7]. PFT are mostly found unilaterally [8].

Double teeth are commonly reported in the anterior region and most often include mandibular lateral incisors and canines [9]. Diagnosis of this phenomenon mainly depends on the case history, as well as the clinical and radiographic examinations.

Clinically, PFT can present a crown of double size or a bifid crown, or have a normal tooth size. Radiographically, their expression can range from two separate roots to a single root depending on the developmental phase of the fused teeth buds. However, clinically, geminated teeth appear as two fully or partially separated crowns, and radiographically, they result in a single root and root canal [10].

A variety of clinical complications have been reported in PDT, including caries formation in the groove between the fused crowns, tooth impaction, malocclusions, and esthetic problems [9].

Currently, three treatment options are suggested for PDT, involving preventive measures, a restorative approach after caries excavation, or a surgical procedure (extraction) [11].

Many studies have illustrated the impact of fused primary teeth on permanent dentition, including aplasia, hyperodontia, and double teeth formation [4, 9].

The present report describes the management of two cases, a first case of unilateral gemination in the primary maxillary left central incisor in a six-year-old boy and a second one of unilateral fusion of a supernumerary tooth with a primary mandibular left lateral incisor in a six-year-old girl.

## 2. Case Presentation

### 2.1. Case Report 1

A 6-year-old Tunisian boy presented to the Pediatric and Preventive Dentistry Department at the Faculty of Dental Medicine of Monastir with a chief complaint of dental caries on the primary maxillary left central incisor (#61) and the maxillary left lateral incisor (#62).

The patient's medical history was unexceptional, and there was no family history of dental abnormalities and no parental consanguinity. No history of trauma was recorded.

The patient reported an occasional hypersensitivity to thermal stimulation (cold and heat) and to sweet in the two anterior carious teeth.

General and extraoral examinations appeared to be noncontributory.

**Figure 1:**
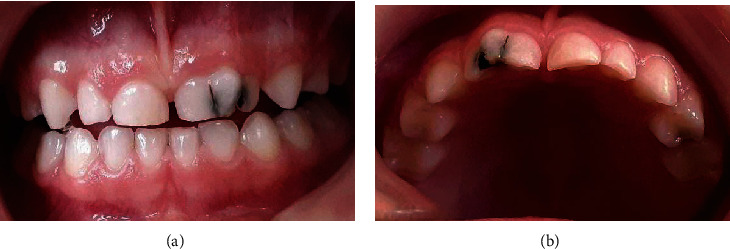
Intraoral photographs. Carious large crown of tooth 61 associated with dental caries of tooth 62 (a). Occlusal photograph showing the anterior teeth nonalignment and distal rotation of tooth 61 (b).

The patient had complete primary dentition. Intraoral examination revealed the presence of a large upper left central incisor (#61) with slightly deep and decayed buccal and lingual grooves and the presence of extensive caries on the distal, lingual, and incisal surfaces of the left lateral incisor (#62) (Figures 1(a) and 1(b)).

Functional examination showed nasal breathing, infant swallowing, and functional chewing.

**Figure 2:**
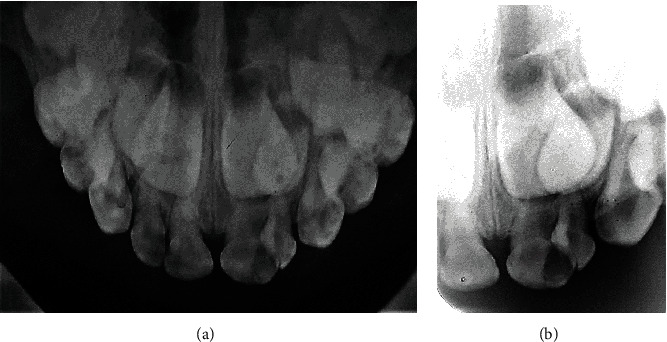
Radiographic examination showing the morphology type: tooth 61 with a single root and a large central root canal. Periapical radiograph (a). Maxillary occlusal radiograph (b).

Periapical radiograph and maxillary occlusal radiograph exhibited the presence of a single large central root canal in the geminated tooth. The carious lesions in both teeth 61 and 62 were extensive and near the pulp chambers (Figures 2(a) and 2(b)).

**Figure 3:**
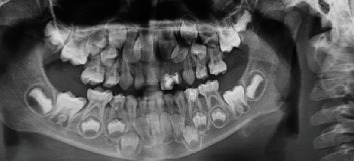
Panoramic radiograph showing the morphology of tooth 22 with a slight developmental delay and rotation.

Panoramic radiograph showed normally erupting permanent incisors, except for the permanent upper left lateral incisor (#22) which exhibited a rotation and a slight developmental delay compared to its homologous tooth (#12) (Figure 3).

Diagnosis of gemination rather than fusion of the maxillary left central incisor (#61) was based on the presence of a complete dentition and on the radiography showing the presence of one root canal for the tooth. Since the patient's chief complaint was carious lesions of the anterior geminated tooth and its contiguous tooth, our decision was to adequately restore the affected teeth.

A detailed explanation was given to the patient's parents regarding the treatment approach. Written informed consent was obtained from the parents, both for the treatment procedure and for the publication of the case report.

Removal of caries was performed using a round carbide metal bur mounted on a low-speed contra-angle (Dentsplay Maillefer, Ballaigues, Switzerland) under local anesthesia and rubber dam isolation. As a pulp exposure occurred, the coronal pulp was tenderly excised with a round bur (Dentsplay Maillefer, Ballaigues, Switzerland) on a slow-speed handpiece (NSK Pana-Max, USA).

**Figure 4:**
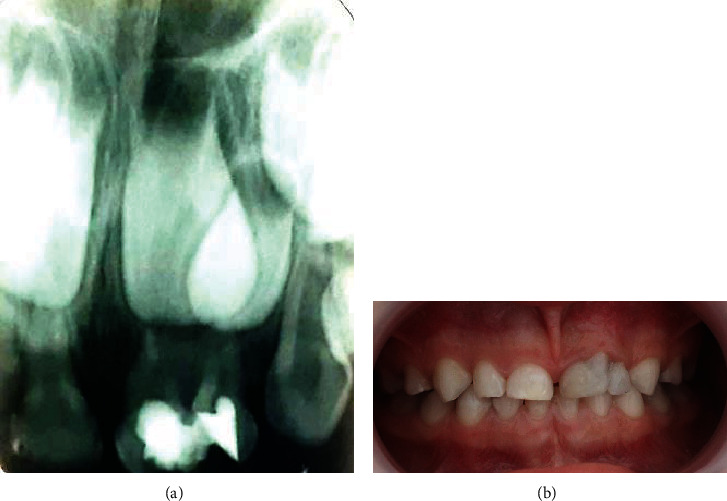
Treatment of teeth 61 and 62. Periapical radiograph showing pulpotomy (a). Coronal restoration (b).

Hemostasis was achieved by the application of sterile cotton pellets for 3 minutes. Then, a cotton pellet was soaked in 2% glutaraldehyde solution, and it was applied in the pulp chamber for 10 minutes. The cotton pellet was later removed, and the pulp chamber was filled with a thick paste of zinc oxide eugenol (ZOE) (Element Zinc oxide eugenol temporary filling material, USA). One week later, the two teeth were asymptomatic, and direct restoration of dental crowns with composite resin (3M ESPE® Restorative Universal Composite A2 and B2) was performed (Figures 4(a) and 4(b)).

The patient was satisfied with the functional and esthetic rehabilitation of the anterior teeth.

A regular 3-month follow-up program was ensured to control the development of tooth 22 and to confirm the possibility of deformation or dysmorphia.

**Figure 5:**
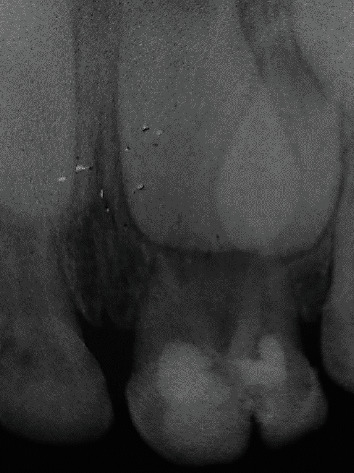
Periapical radiograph of the geminated tooth 61 and its contiguous tooth 62 at the 9-month follow-up visit: the presence of sealed coronal restoration of the two primary teeth with no pathological signs.

At the 9-month follow-up appointment, the tooth was esthetically stable with no pathological clinical signs. The follow-up periapical radiograph showed a perfectly sealed coronal restoration of teeth 61 and 62 (Figure 5).

### 2.2. Case Report 2

A six-year-old Tunisian girl was referred to the Pediatric and Preventive Dentistry Department at the Faculty of Dental Medicine of Monastir with a chief complaint of dental caries in the primary mandibular left canine (#73).

The patient's medical history was unremarkable, and there was no family history of dental anomalies. No consanguinity was reported, and the patient did not undergo any dental trauma.

General and extraoral examinations seemed noncontributory.

Intraoral examination revealed a mixed dentition with the absence of tooth 71 and the presence of dental caries on the gingival third of the labial surface of tooth 73.

**Figure 6:**
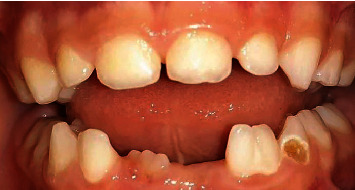
Fusion of the clinical crown of tooth 72 with a supernumerary tooth 72'.

The contiguous primary mandibular left lateral incisor (#72) had a large crown and deep grooves on the labial and lingual surfaces (Figure 6). Examination of occlusion exhibited an anterior open-bite related to the thumb sucking habit. Functional examination revealed dysfunctional chewing, infant swallowing, and nasal breathing.

**Figure 7:**
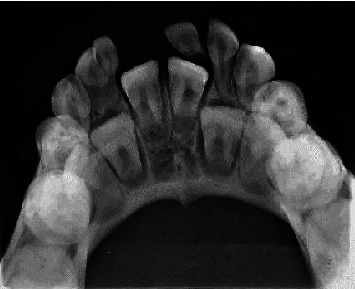
Anterior occlusal radiograph of the mandibular arch showing a type-4 morphology and an incomplete fusion of tooth 72 with a supernumerary tooth.

Anterior occlusal radiograph of the mandibular arch revealed a double tooth type IV morphology since separate pulp chambers and root canals with two distinct but joined roots were observed (Figure 7).

By calculating the number of teeth on the dental arch and the previous exfoliation of tooth 71, it was concluded that the malformation was the result of the fusion of tooth 72 with a supernumerary tooth #72'.

A detailed explanation was given to the patient's parents who signed a written consent for the treatment process and the publication of the case report.

Caries excavation was performed on tooth 73 using a round carbide metal bur mounted on a low-speed contra-angle (Dentsplay Maillefer, Ballaigues, Switzerland) and rubber dam isolation under local anesthesia.

After the application of 2% chlorhexidine cavity disinfectant (Consepsis Ultradent, USA), restoration of tooth 73 using composite resin (3M ESPE® Restorative Universal Composite A2 and B2) was performed.

**Figure 8:**
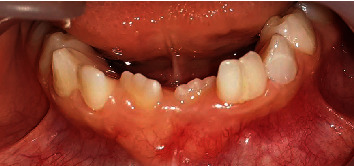
Placement of sealants in the groove of the fused tooth 72 and restoration of the tooth 73.

Preventive measures were applied for the asymptomatic noncarious fused tooth. They included the restoration of the deep grooves using flowable composite (PRIME-Dent® flowable composite A2) (Figure 8). Follow-up visits were scheduled every 3 months.

**Figure 9:**
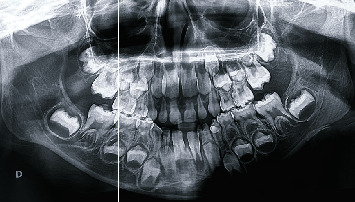
Panoramic radiograph showing the absence of dental abnormalities in the permanent teeth.

A panoramic radiograph was performed after the clinical management of teeth #72 and #73. It showed the presence of all the mandibular permanent teeth without any abnormal signs (Figure 9).

The patient was satisfied with the restoration provided, and she was informed that tooth #72 with a large crown would soon exfoliate.

The patient was also given an interceptive treatment using a functional education device. Recommendations to stop the thumb sucking habit to manage the open-bite and to correct the dysfunctions were also provided.

**Figure 10:**
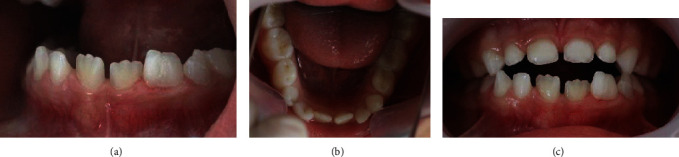
Intraoral photographs of the fused tooth 72 at the 6-month follow-up visit. Stable state with no pathological problems in the two teeth (72 and 73) (a). Occlusal view revealing the anterior teeth proper alignment (b). Photograph of occlusion showing the patient's anterior open-bite (c).

At the 6-month follow-up visit, the fused tooth was still caries-free, and the two permanent mandibular central incisors erupted normally without any dental crowding issues (Figures 10(a)–10(c)).

## 3. Discussion

PDT represent 75% of primary teeth dental anomalies, with fusion accounting for 94% of cases and gemination accounting for 6% of cases [9].

The differential diagnosis of fusion includes gemination and macrodontia [12].

Mader's “two tooth” rule [13] can be a practical method for differentiating between fusion and gemination.

In fact, if the abnormal tooth is considered as “two teeth” and the total number of teeth in the dental arch is normal, fusion is therefore determined. However, when the atypical tooth is considered as “two teeth” and the resulting number of teeth in the dental arch is higher than usual, then, the diagnosis is either gemination or fusion between normal and supernumerary teeth.

Another differentiating point with regard to the two abnormalities is that the fusion between a regular and supernumerary tooth exhibits two distinct halves of the united crown whereas in the case of gemination the two halves of the united crown appear as mirror images [12]. Macrodontia is defined as an abnormal increase in the tooth size with a usual crown, root, and pulp anatomy. Unlike gemination, macrodontia does not present a bifid crown and deep grooves [12].

PDT with deep grooves are more susceptible to dental caries and periodontal problems compared to nonaffected teeth. Those fissures facilitate plaque accumulation and dental caries formation as they may be hard to brush [9]. Therefore, according to the clinical situation, three treatment modalities are available, including preventive measures, a restorative approach, and a surgical procedure [11].

Intact and asymptomatic PDT commonly require a preventive approach, involving local fluoride application, early placement of fissure sealants in the deep grooves, and regular 3-month follow-up program. Dietary changes are also recommended. When a primary double tooth presents a carious lesion, it should be restored with composite resin. In case of trauma or esthetic concerns as seen in maxillary anterior double teeth, guided composite resin restoration using putty silicone is recommended [11].

The third solution is the extraction of the abnormal tooth. This treatment modality can be considered when deep caries lesions, exacerbating tooth mobility or root resorption, are diagnosed. When extraction is performed, space maintenance should be provided when necessary [11]. Regarding periodontal issues, strict oral hygiene is required.

The specificity of the current cases can be due to the multiple effects of PDT on the primary dentition and the repercussions on the permanent successors.

In fact, PDT may cause malocclusions like dental diastema if the fusion occurs between two normal teeth. They may also cause dental crowding if the fusion occurs between a normal tooth and a supernumerary tooth [14].

Reduction in the arch length, deviation of the midline, and esthetic problems due to the irregular morphology may also occur in PDT [9, 15].

Thus, restorative, periodontal, cosmetic, and orthodontic concerns may be necessary to manage the different problems.

In the first case report, distal rotation of the geminated primary maxillary left central incisor (#61), nonalignment of the primary maxillary anterior teeth, and esthetic issues were noticed.

However, in the second case, the patient presented an anterior open-bite related to the thumb sucking habit, not to the fused mandibular tooth.

In the study of Aydinbelge et al. [9], dental abnormalities on the permanent successors were found in 69.4% of the children with PDT. Dental anomalies, such as aplasia, hyperodontia, hypodontia, double tooth formation, peg-shaped teeth, talon cusp, and delayed eruption generally related to odontoma formation have been reported in succedaneous teeth [9, 16].

In the first case, when compared with its contralateral incisor (#12), a rotation and a slight developmental delay in the permanent left lateral incisor (#22) were noticed. However, a long-term monitoring is necessary to confirm this investigation as all the permanent maxillary incisors are still in development. Moreover, the patient's age should be taken into account.

In the second case report, the 6-month follow-up visit showed proper alignment of the anterior mandibular permanent teeth. However, interceptive treatment to manage the open-bite and oral dysfunctions was scheduled.

Follow-up visits every 3 months at the Pediatric and Preventive Dentistry Department were scheduled to control the permanent teeth development.

Many factors impact the prognosis of PDT, involving the tooth condition and the associated complications, such as dental caries and malocclusions. When a primary double tooth does not have any indication to be extracted, prognosis is usually favorable [11].

In the two cases, preventive, restorative, and regular monitoring were chosen as the two double teeth were restorable.

Finally, to manage this developmental disorder successfully requires an early diagnosis, a convenient choice of the treatment modality, and a regular 3-month follow-up program. It is also recommended to determine the presence of any dental anomaly in the permanent successors.

## 4. Conclusion

PDT are one of the most frequent dental anomalies in primary dentition.

Clinical and radiographic examinations are required in the diagnosis of PDT, and a multidisciplinary treatment is necessary in some cases.
